# Case report: the dissociated response and clinical benefit of primary leiomyosarcoma of the bone treated with penpulimab plus lenvatinib after failed multi-line therapy

**DOI:** 10.3389/fphar.2023.1239699

**Published:** 2023-11-09

**Authors:** Bin Wang, Yin Han, Jie Liu, Xinyao Zhang, Hongyu Zhuo, Yu Jiang, Yaotiao Deng

**Affiliations:** ^1^ Medical Oncology, Cancer Center, West China Hospital, Sichuan University, Chengdu, China; ^2^ Department of Pathology, Cancer Prevention and Treatment Institute of Chengdu, Chengdu Fifth People’s Hospital, The Second Clinical Medical College, Affiliated Fifth People’s Hospital of Chengdu University of Traditional Chinese Medicine, Chengdu, China

**Keywords:** leiomyosarcoma, immunotherapy, anti-angiogenic therapy, dissociated response, clinical benefit

## Abstract

Leiomyosarcoma occurring in the bone as primary tumor localization is extremely scarce with limited cases described in the literature, accounting for less than 0.7% of all primary bone malignancies. Once distant metastasis occurs, patients have limited treatments and often a somber prognosis, which underscore the need for innovative and effective treatment approaches. The emerging evidence suggests that anti-angiogenic therapy could inhibit angiogenesis and normalize vascular permeability in the tumor microenvironment, which, in turn, would increase immune effector cell infiltration into tumors. Immunotherapy depends on the accumulation and activity of immune effector cells within the tumor microenvironment, and immune responses and vascular normalization seem to be reciprocally regulated. Immunotherapy combined with anti-angiogenic therapy has recently made great progress in the treatment of various types of tumors. However, the effectiveness of the combination treatment in metastatic leiomyosarcoma is undetermined. In this study, we presented a rare case of primary leiomyosarcoma of the bone located in the trochanteric region of the femur, accompanied by multiple distant metastases. After the failure of multi-line therapies including AI regiments as the adjuvant chemotherapy, anlotinib as the first-line therapy, GT regiment as the second-line therapy, and eribulin as the third-line therapy, the patient received combinational therapy with penpulimab plus lenvatinib. The best efficacy for this regimen was a partial response, with a progression-free survival of 8.4 months according to the iRECIST criteria. After a dissociated response was detected without severe toxicities, the patient received local radiotherapy and continued treatment on penpulimab plus lenvatinib and eventually achieved long-term survival benefits with a total of over 60 months of overall survival with good quality of life and ongoing treatment. As our previous retrospective study found that one-third of advanced STS patients could still achieve clinical benefits from rechallenge with multi-targeted tyrosine kinase inhibitors (TKIs), after the failure of previous TKI therapy, this case provided the potential clinical activity of immunotherapy combined with anti-angiogenic TKI rechallenge in metastatic leiomyosarcoma.

## Introduction

Leiomyosarcoma (LMS) is a malignant neoplasm derived from either smooth muscle cells or precursor mesenchymal stem cells that eventually differentiate into smooth muscle cells, with an estimated incidence of 10%–20% of all newly diagnosed soft tissue sarcomas (STSs) (Novotny and George, 2021). This tumor can originate from any site of the body, with the retroperitoneum, uterus, and limbs/girdles being more frequently affected (George et al., 2018). LMS that occurred in bones as primary tumor localization is extremely rare and was first reported by Evans and Sanerkin in 1965, with an incidence of less than 0.7% of all primary bone malignancies (Evans and Sanerkin, 1965; Wang and Lucas, 2019). Primary leiomyosarcoma of the bone (PLMSB) usually occurs in the long tubular bones of the lower extremities, with about 70% of cases found in the knee joint (distal femur and proximal tibia) (Wu et al., 2022). It is rarely reported to occur in the hip joint and trochanteric area of the femur. The diagnosis of PLMSB is marked by the absence of either osteoid or chondroid matrix LMS. Clinical features and relevant prognostic factors of PLMSB are not well defined because of the few data obtained mostly from retrospective analyses, small case series, and case reports (Wang and Lucas, 2019). LMS has a predilection for metastasis, with common sites of metastasis including the lung, liver, soft tissues, and bones; metastasis to the pancreas is extremely rare (Rekhi et al., 2011).

Systemic chemotherapy with doxorubicin alone or in combination is still the first-line treatment for unresectable metastatic LMS (Pautier et al., 2022). Despite multiple clinical trials investigating single-agent and combination schemes over the past decades, progression-free survival (PFS) for various therapies remains in the 3–7-month range with median overall survival (OS) at 12–18 months (Miller et al., 2020). Second- and later-line regimens provide small clinical benefits in patients with STSs including LMS (e.g., pazopanib, trabectedin, and eribulin). LMS is a disease of complex cytogenetic and molecular aberrancies and is characterized by a relatively inflamed TME with higher PD-L1 expression, greater immune infiltration, and antigen presentation compared with other sarcoma subtypes (Feng et al., 2023; Lacuna et al., 2023). Despite the potential for immunotherapy in LMS, immune checkpoint inhibitors (ICIs) targeting PD-1, PD-L1, and CTLA-4 have greatly improved oncologic outcomes for several cancer types but show minimal efficacy for LMS (Tawbi et al., 2017). Thus, treatment regimens with higher efficacy are needed to improve the outcomes of patients with metastatic LMS. Immune barrier mediated by tumor angiogenesis is well established, and there is an ever-growing list of immune cells exhibiting the dual capacity of facilitating angiogenesis and immunosuppression (Fukumura et al., 2018). Through directly inhibiting tumor growth and metastasis, anti-angiogenic therapies reprogram the tumor milieu from an immunosuppressive to an immune-permissive microenvironment, which, in turn, increases immune effector cell infiltration into tumors (Lee et al., 2020). Activated immunity by ICIs can also enhance anti-angiogenesis by decreasing the expression of VEGF and alleviating hypoxia (Lee et al., 2020; Wang et al., 2023). ICIs combined with anti-angiogenic therapy have exhibited favorable outcomes in various types of cancers (Fukumura et al., 2018). Penpulimab is a novel Fc-engineered IgG1 monoclonal antibody against PD-1. By eliminating fragment crystallizable (Fc) receptor-binding activities such as antibody-dependent cell-mediated cytotoxicity (ADCC) and antibody-dependent cellular phagocytosis (ADCP), the agent not only enhances the efficacy of immunotherapy but also exhibits improved safety profiles. Lenvatinib, an oral multi-targeted tyrosine kinase inhibitor (TKI), has potent anti-angiogenic activity, mainly through the inhibition of VEGFR 1–3, PDGFRα, FGFR 1–4, KIT, and RET. To date, little information is available on the efficacy and safety of this combination in metastatic LMS.

Here, we report a rare case of leiomyosarcoma metastatic to the pancreas in a patient with confirmed PLMSB in the trochanteric region of the femur. After progressing on third-line therapy, the patient received the fourth-line therapy with penpulimab plus lenvatinib and eventually achieved long-term benefits with a total of over 60 months of overall survival with a good quality of life and ongoing treatment. This case demonstrated the efficacy and safety of immunotherapy combined with anti-angiogenic therapy for the later-line treatment of metastatic PLMSB and provided the possibility of a rechallenge with TKI.

## Case presentation

**FIGURE 1:**
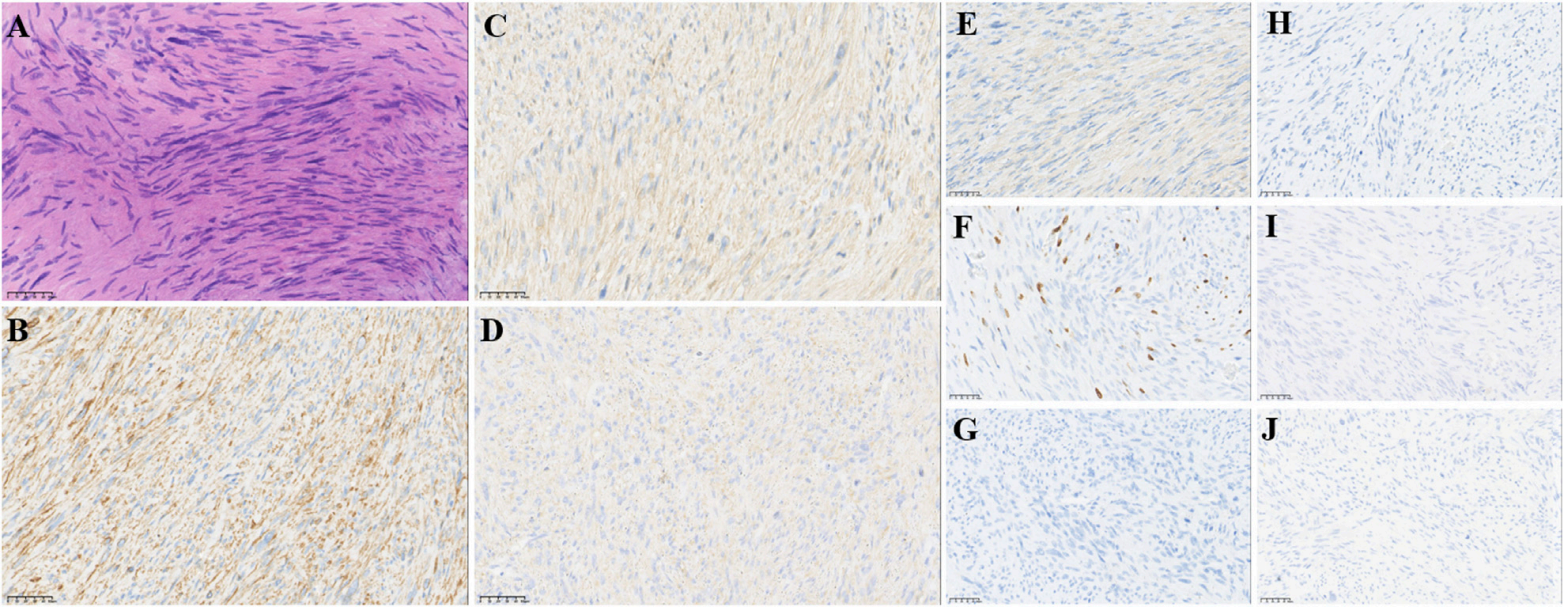
Pathological findings of post-operative biopsies. **(A)** HE staining revealed spindle cell morphology (10 ×), IHC staining showed that lymphoma cells were positive for **(B)** desmin, **(C)** calponin, **(D)** caldesmon, **(E)** SMA, and **(F)** MIB1 and negative for **(G)** S100, **(H)** CR, **(I)** NF, **(J)** and SATB2, supporting the diagnosis. Original magnification: **(A–J)**, 200 ×.

**FIGURE 2:**
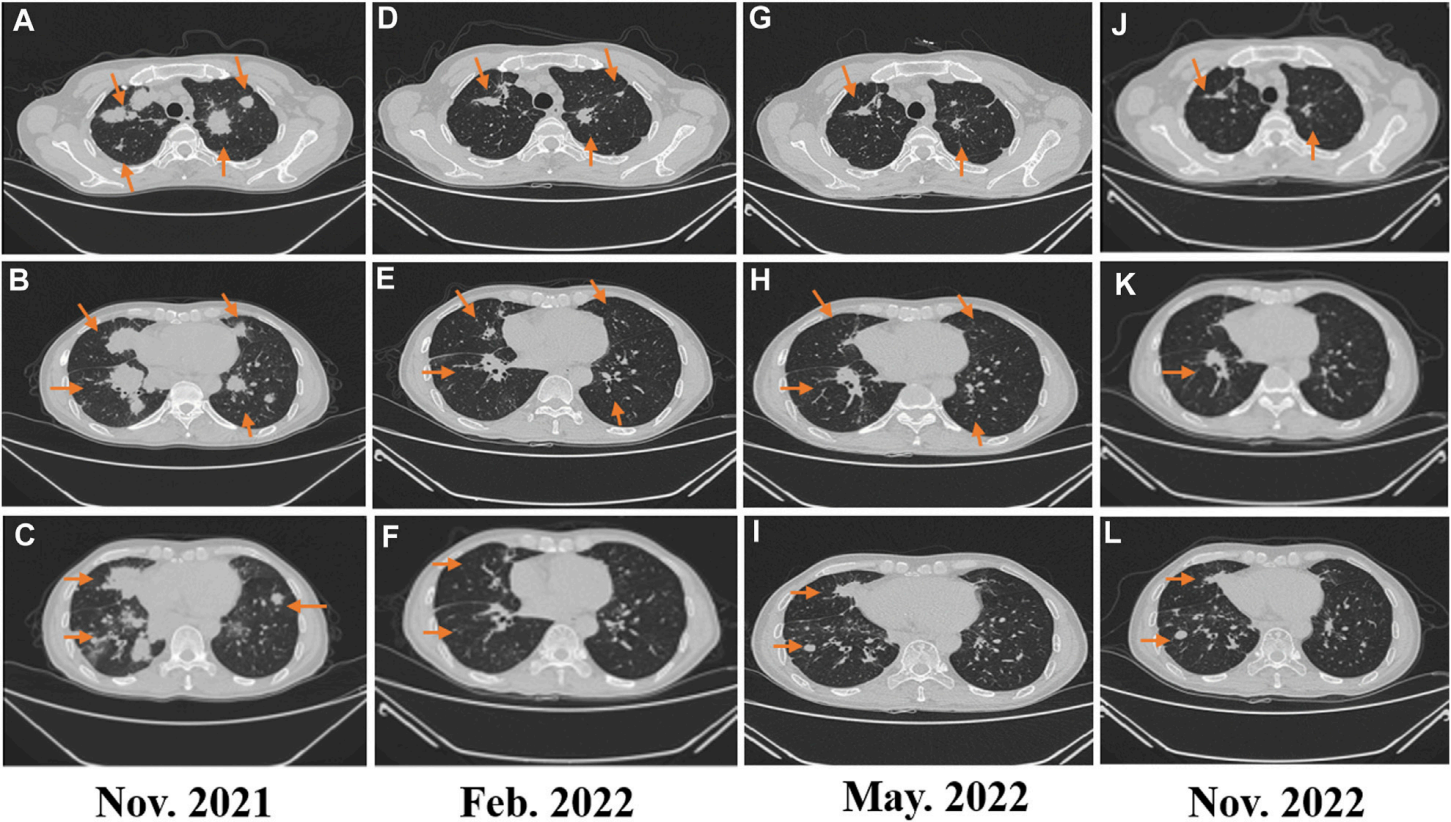
**(A–C)** Image evaluation after second-line treatment with eribulin for two cycles: progressive disease; **(D–E)** image evaluation after fourth-line treatment of penpulimab combined with lenvatinib in February 2022: progressive disease; **(G–I)** image evaluation after fourth-line treatment of penpulimab combined with lenvatinib in May 2022: dissociated response; and **(J–L)** image evaluation in November 2022: dissociated response.

**FIGURE 3:**
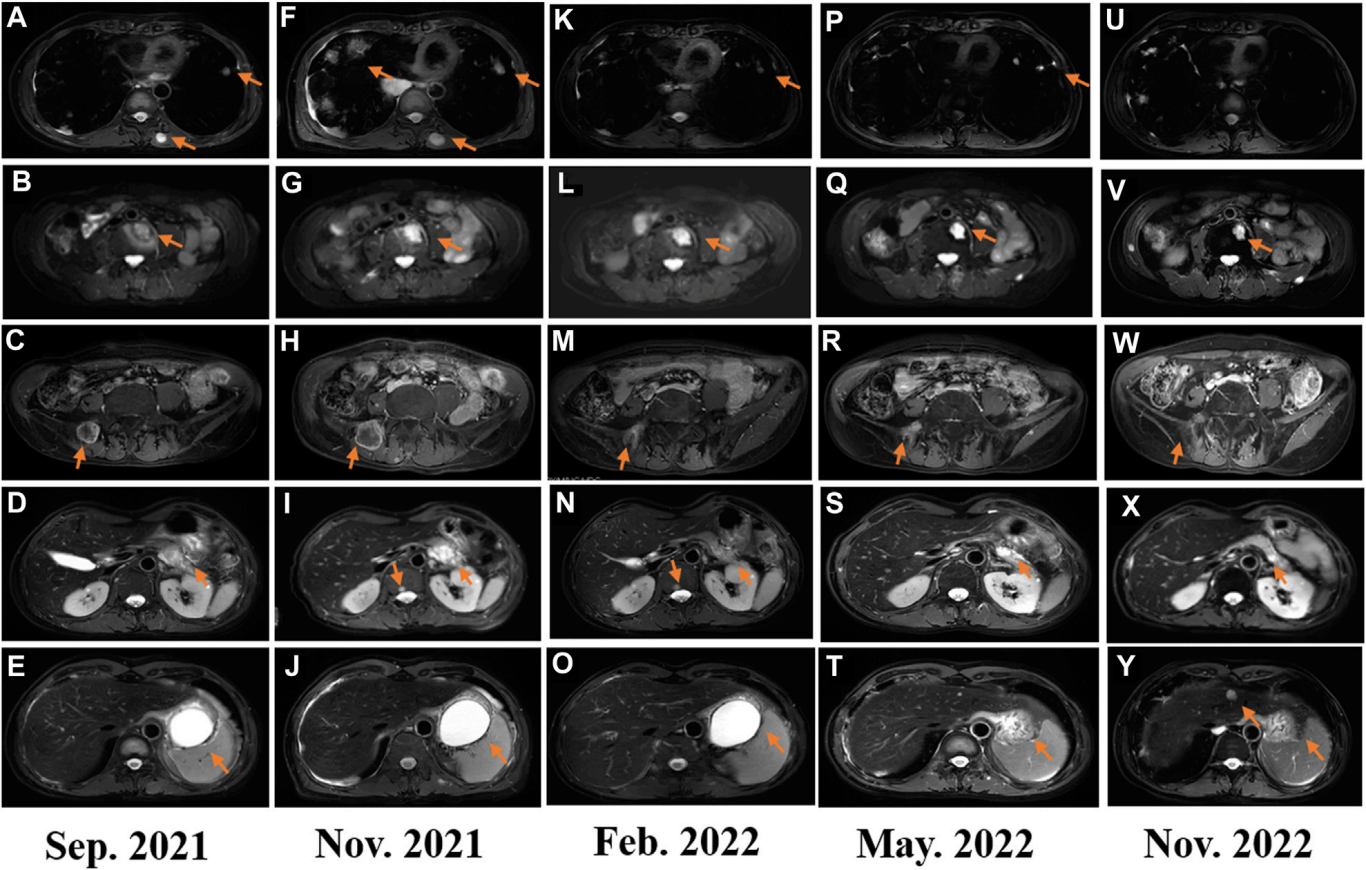
**(A–F)** Image evaluation after second-line treatment of gemcitabine plus docetaxel in September 2021: progressive disease; **(F–J)** image evaluation after third-line treatment with eribulin for two cycles: progressive disease; **(K–O)** image evaluation after fourth-line treatment of penpulimab combined with lenvatinib in February 2022: progressive disease; **(P–T)** image evaluation after fourth-line treatment of penpulimab combined with lenvatinib in May 2022: dissociated response; and **(U–Y)** image evaluation in November 2022: dissociated response.

**FIGURE 4:**
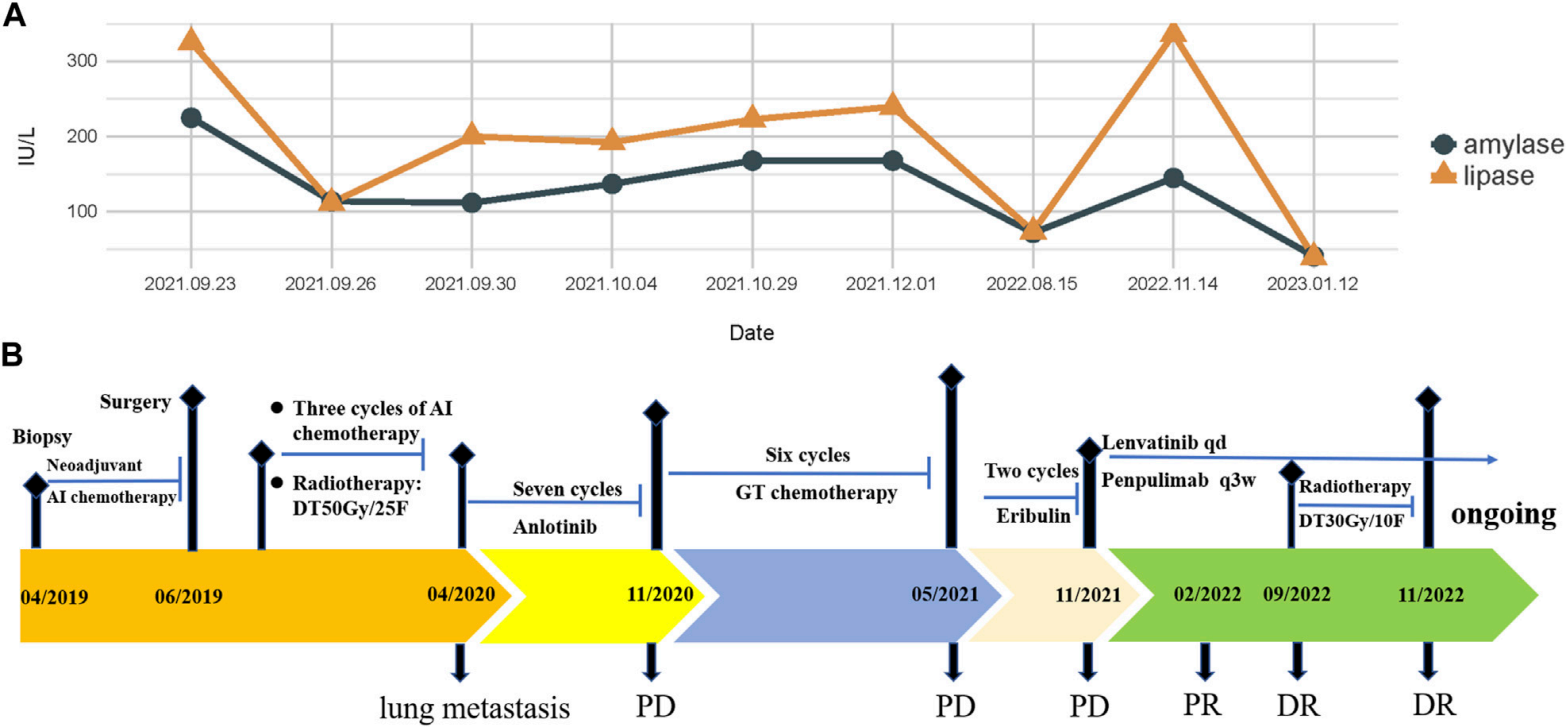
**(A)** Changes in serum amylase and lipase levels during the treatment; **(B)** treatment timeline of the patient with PLMSB.

In February 2018, a 49-year-old male presented with an enlarged palpable mass in the proximal right lower extremity, accompanied by persistent dull pain and movement restriction. The patient did not have any previous significant medical conditions. The computed tomography (CT) revealed the shadow of a heterogeneous mass (89 × 42 mm) on the right intertrochanteric region of the femur , and the lesion was obviously strengthened unevenly after enhancement. Metastatic tumors were excluded according to the patient’s whole-body imaging findings. In August 2018, an excisional biopsy of the right intertrochanteric space-occupying lesion was performed, and the pathological evaluation demonstrated a right femoral intertrochanteric leiomyosarcoma. After two cycles of neoadjuvant doxorubicin–ifosfamide (AI) chemotherapy comprising doxorubicin 75 mg/m^2^ given at day 1 and ifosfamide at 1.8 g/m^2^ per day over 5 days every 3 weeks, the patient underwent extensive resection of femoral intertrochanteric leiomyosarcoma and total hip arthroplasty under general anesthesia on 5 December 2018. Macroscopic examination of the resected bone segment showed a grayish-yellow mass with a size of 8 cm × 7 cm × 6 cm in the bone marrow cavity and 9 cm from the broken end of talus tissue, which had a pattern of growth replacing the marrow and invading the surrounding soft tissues. The efficacy assessment of neoadjuvant chemotherapy revealed extensive necrosis in the tumor with a tumor necrosis rate of approximately 80%–90% compared with preliminary pathologic results. The final pathology of the post-operative specimens suggested spindle cell morphology with immunohistochemical results being positive for desmin, calponin, caldesmon, SMA, and MIB1 and negative for S100, CR, NF, and SATB2 and confirmed the diagnosis of left femoral intertrochanteric leiomyosarcoma, grade III (FNCLCC) (Figure 1). Three cycles of adjuvant AI regimen chemotherapy were given after surgery, followed by field adjuvant radiotherapy at a dose of 50 Gy until May 2019. Unfortunately, multiple newly bilateral pulmonary nodules were detected by the routine chest CT scan on April 2020 (Supplementary Figures S1A–D). Subsequently, the patient underwent first-line treatment with anlotinib (12 mg, d1–14) every 3 weeks for seven cycles from June to October 2020 and achieved a PFS of 4 months. Then, the number and size of bilateral pulmonary nodes increased, and the curative effect was evaluated as progression disease (PD) based on the RECIST criteria (Supplementary Figures S1E–H). Thus, the patient accepted second-line chemotherapy of gemcitabine at the dose of 1,000 mg/m^2^ on days 1 and 8, combined with docetaxel at the dose of 75 mg/m^2^ on day 8 for six cycles. After a PFS of 8 months, the patient began complaining of persistent epigastric abdominal sharp pain with the numeric rating scale score of 7, accompanied by fever, nausea, vomiting, abdominal distension, and diarrhea in May 2021. The patient was not presented with melena, swallowing difficulties, or shortness of breath. He was bedridden for the vast majority of the time with an ECOG performance status score of 3, which severely compromised his life quality. The follow-up imaginological examination revealed new metastases in bilateral erector spinae, left internal abdominal oblique muscle, L1 and L4 vertebra, and pancreas, along with acute necrotizing pancreatitis and pancreatic pseudocyst (Figures 3A–E). At that time, the laboratory examination showed the abnormal elevation of serum amylase (up to 182 IU/L) and lipase (up to 325.4 IU/L) (Figure 4A). After broad-spectrum antibiotics and other supportive treatments, the patient’s abdominal pain was significantly alleviated, and the levels of both serum amylase and lipase gradually declined but remained persistently above the normal reference range (Figure 4A). Subsequently, the patient began to try third-line therapy with eribulin at the dose of 1.4 mg/m^2^ administered intravenously on days 1 and 8 every 21 days from September 2021. However, the patient did not benefit from eribulin, with the disease being judged to progress after only two cycles (Figures 2A–C, 3F–J). In December 2021, the patient received the combined treatment with penpulimab (200 mg IV infusion every 3 weeks), a humanized anti-PD-1 IgG1 antibody, plus the multikinase inhibitor lenvatinib (8 mg/day). The accompanying treatment plan consisted of denosumab (120 mg) injections every 4 weeks to prevent skeletal-related events. After two cycles, the physical conditions and life quality of the patient had improved significantly, with pain fading away, weight restoration, and the ECOG performance status score improving to 1. The level of serum amylase and lipase demonstrated a constant downward trend with complete normalization (14.39 U/mL) by the end of his six cycles of treatment (Figure 4A). A CT re-examination in February 2022 indicated that tumors had shrunk along with the absorption of the inflammatory exudation around the pancreas and the recession of the pancreatic pseudocyst, and the efficacy evaluation was partial response (PR) (Figures 2D–F, 3K–O). The PFS for this regimen was 8.4 months according to the iRECIST criteria.

Interestingly, repeated CT and MRI scans in May 2022 pictured a mixed response with a new pulmonary lesion observed in the posterior basal segment of the right lung but a regression in the size of the rest of the target lesions, which suggested an immune-unconfirmed progressive disease (iUPD) according to the iRECIST criteria. In August 2022, the previous new nodule of the lung continued to grow, but the rest of the lesions remained stable, which indicated immune-confirmed progressive disease (iCPD), with a pattern of dissociated response (DR) (Figures 2G–I, 3P–T). Given the clinical benefit of previous combination therapy, after a multi-disciplinary treatment (MDT) discussion and a thorough risk communication with the patient, he was treated with radiotherapy for the new pulmonary lesion (30 Gy in 10 fractions) in September 2022 while continuing penpulimab plus lenvatinib treatment. The latest imaging assessment in November 2022 revealed that all lesions were all reduced or stable, with the exception of a new metastasis in the right lobe of the liver (Figures 2J–L, 3U–Y). The patient was planned to be treated with SBRT for the hepatic metastatic lesion. Until now, the patient is still alive and undergoes treatment with lenvatinib plus penpulimab, with the OS time being extended to over 60 months and counting. Importantly, during the course of combination therapy, he had no other adverse events related to anti-angiogenic therapy or immunotherapy except hypothyroidism and osteoarthritis, which were controlled well by levothyroxine sodium tablets and short-term treatment of prednisone, respectively. In order to intuitively reflect the patient’s prognosis and clinical curative effect, we listed the entire treatment process with timeline in Figure 4B.

## Discussion

Primary LMSB is a rare and highly invasive leiomyosarcomas, first described in 1965, with an incidence of less than 0.7% among all primary bone malignancies (Wang and Lucas, 2019). PLMSB generally has non-specific clinical and radiologic presentations (Wang and Lucas, 2019). The histopathologic features of PLMSB are identical to those originating from other more prevalent anatomical sites, characterized by smooth muscle differentiation and the absence of malignant osteoid formation (Wang and Lucas, 2019). The diagnosis of PLMSB is a challenge, which must be distinguished from metastatic leiomyosarcoma from other sites, fibroblastic osteosarcoma, primary undifferentiated pleomorphic sarcoma (UPS) of bone, and metastatic sarcomatoid carcinoma (George et al., 2018). Fibroblastic osteosarcoma tissues are arranged as fibrosarcomatoid structures, and the identification of focal malignant osteogenesis and the absence of myogenic tumor markers aid in distinguishing osteosarcoma from PLMSB (Wang and Lucas, 2019). In addition, SATB2 is a relatively specific marker for osteosarcoma that may be utilized to differentiate between osteosarcoma and PLMSB (Wang and Lucas, 2019). UPS is typically an exclusion diagnosis, and myogenic markers such as desmin and SMA can be particularly valuable in differentiating UPS from PLMSB (Matushansky et al., 2009). Metastatic sarcoma is morphologically similar to PLMSB but has unique immunohistochemical profiles, with the expression of p63 and PAX8 and lack of myogenic markers like desmin and SMA (Wang et al., 2021). The lesion reported in this case presented as a spindle cell sarcoma by microscopy, and immunohistochemistry showed positive staining for desmin, calponin, caldesmon, SMA, and MIB1 and negative staining for S100, CR, NF, and SATB2. Metastatic tumors were excluded according to the patient’s medical history and whole-body imaging findings. Based on the absence of malignant osteogenesis and positive myogenic markers, diseases mentioned previously were ruled out, and a definite diagnosis of PLMSB in the right intertrochanteric region of the femur was finally reached.

Patients with PLMSB tended to have a worse prognosis than those with soft tissue LMS (Kobayashi et al., 2022). Due to its low prevalence, data on optimal treatment for PLMSB are limited. When possible, surgery with negative surgical margins remained the primary treatment modality, independent of origin sites (Wang and Lucas, 2019). No clear survival benefit has been demonstrated with the use of adjuvant chemotherapy or radiotherapy (Zhang et al., 2022). However, due to possible differences in biological behavior, chemotherapy and/or radiotherapy are still recommended, especially for cases that failed to completely resect the lesion (Sun et al., 2022). Local recurrence is relatively uncommon, while distant metastases are more likely to develop early, generally in the first year, regardless of the initial tumor grade (Rekhi et al., 2011). The most frequent metastatic sites are the lung and the axial skeleton, while metastases to the pancreas are not common and few cases have been reported (Rekhi et al., 2011). In our case, the patient developed multiple metastases including the pancreas within 8 months after extended resection and adjuvant AI regimen chemotherapy and radiotherapy. For advanced STS/LMS, anthracycline‐based chemotherapy is the cornerstone therapy, with a median overall survival of approximately 1 year (Novotny and George, 2021). After the failure of first-line chemotherapy, there is no evidence to indicate which regimen is optimal for second-line therapy and beyond. Some chemotherapeutic agents like gemcitabine, eribulin, and trabectedin have exhibited certain efficacy for LMS, but their significance in improving OS remains controversial (Meyer and Seetharam, 2019). Some tyrosine kinase inhibitors, including pazopanib, regorafenib, and sunitinib among others, have demonstrated efficacy in STS/LMS. Moreover, multi-targeted TKI anlotinib against VEGFR1, VEGFR2/KDR, VEGFR3, FGFR1-3, PDGFR-α, and c-Kit has been licensed in China for the treatment of advanced STS after the failure of anthracycline-based chemotherapy based on phase II (Chi et al., 2018a) and phase IIb studies (ALTER0203) (Chi et al., 2018b). The subgroup analysis from a phase III study in LMS patients demonstrated the superior efficacy of eribulin compared to dacarbazine, with a median PFS of 2.2 months versus 2.6 months, a median OS of 12.7 months versus 13.0 months, and an ORR of 5% versus 7%, respectively (Blay et al., 2019). Given these premises, we selected the AI regimen, anlotinib, and GT regimen as adjuvant chemotherapy and first- and second-line treatments for this patient, respectively. The patient experienced the progression of pancreatic metastases that presented as severe acute pancreatitis while receiving second-line therapy. He was managed conservatively with supportive treatment, followed by two cycles of eribulin salvage chemotherapy. Regrettably, the patient did not benefit from single eribulin, with the therapeutic evaluation being PD.

In general, there is little evidence that immunotherapies work for LMS, barring a few case reports. The SARC028 trial revealed that the objective response rate (ORR) was 17.5% among patients with undifferentiated pleomorphic sarcoma (4/10), liposarcoma (2/10), and synovial sarcoma (1/10), while no response was observed in the LMS subgroup (Tawbi et al., 2017). The poor outcome was in line with another phase II study confirming the lack of efficacy of single nivolumab in uterine LMS (Ben-Ami et al., 2017). Many studies have shown that the combination of anti-angiogenic agents and ICI might enhance the presence and activation of CTLs in the TME to further enhance the anti-tumor effect, emerging as a novel treatment strategy (Roulleaux Dugage et al., 2021). A phase 2 trial (NCT02636725) revealed that pembrolizumab plus axitinib had preliminary activity and manageable toxicity in patients with advanced STSs, with a 3-month PFS rate of 65.6% (Wilky et al., 2019). Another phase II trial (NCT03798106) showed encouraging activity in advanced STS, achieving an ORR of 28.3% (Kim et al., 2021). Results of a single-center retrospective analysis enrolling 61 patients with advanced STSs also showed that the median PFS was significantly prolonged after ICI–TKI combination compared to ICI alone, with 50% (8/16) of LMS patients achieving SD (You et al., 2021). A phase 2 trial (NCT04551430) revealed that cabozantinib combined with ipilimumab and nivolumab was superior to cabozantinib for the treatment of non-translocation STSs in DCR and PFS, with LMS being the most frequent responding histology (Van Tine et al., 2023). The patient initiated combinational therapy with penpulimab plus lenvatinib, and the efficacy evaluation was PR after 3 months. Different from other monoclonal antibodies against PD-1 which all use IgG4 subtypes, penpulimab is an IgG1 monoclonal antibody engineered to completely eliminate Fcγ receptor binding and Fc-mediated effector functions that can impair anti-tumor activity, exhibiting better stability, less host cell protein residue, and more favorable safety profiles (Tawbi et al., 2017). Lenvatinib, a multi-targeted TKI inhibitor, has potent anti-angiogenic activity, mainly through the inhibition of VEGFR 1–3, PDGFRα, FGFR 1–4, KIT, and RET [5]. However, another phase 2 trial (NCT04784247) evaluating the efficacy of lenvatinib and pembrolizumab in selected sarcomas suggested there were no responses in the LMS cohort with a poor PFS (Avutu et al., 2023). Due to the intra-/intertumor heterogeneity of LMS, it is not sufficient to guide combination approaches with current biomarkers such as PD-L1, TILs, and TMB. Ectopic lymphoid aggregates, termed as tertiary lymphoid structures (TLSs), have been recently demonstrated to be related to higher response to immunotherapy and superior prognosis in various types of cancers including STSs, independent of CD8^+^ T-cell density and PD-L1 status (Schumacher and Thommen, 2022; Wang et al., 2022). Anti-angiogenic immune-modulating therapies have been proven to induce tumoral high-endothelial venules with T-cell-enriched TLSs, which exhibit an improved tumor response (Hua et al., 2022). The predictive value of TLS in STSs has been validated prospectively. The PEMBROSARC trial suggested a significant improvement in the ORR and 6-month non-progression rate in the TLS-positive STS cohort compared to the previously unselected cohort (Italiano et al., 2022). The SPARTO study (NCT05210413) is ongoing to evaluate spartalizumab combined with pazopanib in solid tumors including TLS-positive STSs. In addition, the CONGRATS study (NCT04095208), still recruiting, includes STS patients with a sarcoma enriched with TLS to evaluate the combination of nivolumab and relatlimab, with results expected in the near future. This case not only proved the superior clinical activity of ICIs and anti-angiogenic therapy but also displayed the possibility of TKI rechallenge with lenvatinib after the failure of anlotinib therapy in advanced LMS. The median time interval between initial TKI treatment and TKI rechallenge was 14 months. The long TKI treatment interval with other types of therapy might change the tumor microenvironment and make tumors restore the sensitivity to anti-angiogenic therapy. Our previous retrospective study also found that 34.6% of advanced STS patients could still achieve clinical benefits from rechallenge with multi-targeted TKI after the failure of previous TKI therapy, with a median OS of 11.7 months and a median PFS of 3.3 months (Liu et al., 2021). Another explanation for the efficacy of TKI rechallenge might be the differences in targets and affinities among the multi-targeted TKIs. Apart from different targets, lenvatinib and anlotinib have different affinities with VEGFR. It is well known that multi-targeted TKI therapy increased the expression of PD-L1 and tumor mutation burden, and TKI-resistant clones made tumor cells more sensitive to combination immunotherapy (Isomoto et al., 2020). It was assumed that the TKI-sensitive clone might re-populate and dominate the tumor cell population after prolonged exposure to ICIs, causing immunotherapy resistance while restoring TKI sensitivity (Lam et al., 2021). The mechanism of TKI efficacy in this rechallenge setting needs further exploration.

After more than 8 months of treatment with penpulimab and lenvatinib, the patient was confirmed to have progressive disease based on the iRECIST criteria but with a pattern of dissociated response (DR). This atypical response pattern is analogous to mixed responses in settings of chemotherapy and targeted therapy, which was defined as the simultaneous coexistence of responding and non-responding lesions within the same patient (Borcoman et al., 2019). The incidence of DR in cancer patients receiving systemic chemotherapy and targeted therapy has been previously reported to range from 13.9% to 39.0% (Guan et al., 2022). DR has also been found in patients receiving combination immunotherapy, like PD-1/PD-L1 inhibitors combined with chemotherapy, targeted therapy, or radiotherapy, with a DR rate of 12.5% in mesothelioma, 13.2% in NSCLC, 14.3% in endometrial carcinoma, and 30.3% in RCC (Guan et al., 2022). To date, the DR rate in STS has been rarely reported in the literature. In contrast to PR/complete response, DR is considered as an unfavorable prognostic factor for patients receiving targeted therapy or chemotherapy. However, in almost all studies regarding the response pattern of immune-related DR, patients with DR had a prolonged OS or increased clinical benefit compared to those who achieved true PD (39). Sato et al. (2021) revealed that DR had significantly longer OS compared to those showing PD (46.9 versus 8.2 months) in advanced NSCLC patients treated with nivolumab. Furthermore, a durable clinical benefit was observed in approximately 20%–50% of patients with DR after the continuation of immunotherapy (Humbert et al., 2020). Importantly, DR cannot simply be considered as a true tumor progression and does not represent real-acquired resistance to ICIs (Borcoman et al., 2019). Although no clear recommendations exist in DR setting, immediately discontinuing immunotherapy or switching to other systematic therapies may not be an early alternative strategy (Borcoman et al., 2019). If possible, local treatments of progressing lesions should be discussed in selected patients with good clinical conditions (Borcoman et al., 2019). Liniker et al. (2016) demonstrated the feasibility of salvage radiotherapy to lesions progressing on the PD-1 blockade in advanced melanoma, achieving an overall response rate of 45%. This patient received local radiotherapy to the single metastatic lesion of the lung and liver, respectively, while continuing treatment with penpulimab plus lenvatinib and eventually obtained a long-term and high-quality survival benefits.

In summary, we presented a rare case of PLMSB in the right intertrochanteric region of the femur . After the failure of multi-line therapies, this case not only proved the superior clinical activity of immunotherapy combined with anti-angiogenic therapy in PLMSB but also showed the possibility of TKI rechallenge. Localized therapy of progressive lesions after DR was an important treatment strategy that could be beneficial to advanced LMS patients. So far as we know, no similar therapeutic regimens and strategies have been reported in PLMSB, and our findings provided new insights into therapeutic options for advanced PLMSB, which still need to be tested in clinical trials with larger samples.

## Data Availability

The original contributions presented in the study are included in the article/Supplementary Material; further inquiries can be directed to the corresponding author.

## Supplementary material

SUPPLEMENTARY FIGURE S1
**(A–D)** Multiple newly bilateral pulmonary nodules were detected by the routine chest CT scan on April 2020; **(E–H)** the progression bilateral pulmonary lesions noted after the first-line treatment with anlotinib for seven cycles (October 2020).

## References

[B1] Avutu V. Chi P. Dickson M. A. Gounder M. M. Kelly C. M. Keohan M. L. (2023). A pilot study of lenvatinib plus pembrolizumab in patients with advanced sarcoma. J. Clin. Oncol. 41 (16_Suppl. l), 11517. 10.1200/jco.2023.41.16_suppl.11517

[B2] Ben-Ami E. Barysauskas C. M. Solomon S. Tahlil K. Malley R. Hohos M. (2017). Immunotherapy with single agent nivolumab for advanced leiomyosarcoma of the uterus: results of a phase 2 study. Cancer 123 (17), 3285–3290. 10.1002/cncr.30738 28440953 PMC5762200

[B3] Blay J. Y. Schöffski P. Bauer S. Krarup-Hansen A. Benson C. D'Adamo D. R. (2019). Eribulin versus dacarbazine in patients with leiomyosarcoma: subgroup analysis from a phase 3, open-label, randomised study. Br. J. Cancer 120 (11), 1026–1032. 10.1038/s41416-019-0462-1 31065111 PMC6738064

[B4] Borcoman E. Kanjanapan Y. Champiat S. Kato S. Servois V. Kurzrock R. (2019). Novel patterns of response under immunotherapy. Ann. Oncol. 30 (3), 385–396. 10.1093/annonc/mdz003 30657859

[B5] Chi Y. Fang Z. Hong X. Yao Y. Sun P. Wang G. (2018a). Safety and efficacy of anlotinib, a multikinase angiogenesis inhibitor, in patients with refractory metastatic soft-tissue sarcoma. Clin. Cancer Res. 24 (21), 5233–5238. 10.1158/1078-0432.CCR-17-3766 29895706

[B6] Chi Y. Yao Y. Wang S. Huang G. Cai Q. Shang G. (2018b). Anlotinib for metastasis soft tissue sarcoma: a randomized, double-blind, placebo-controlled and multi-centered clinical trial. J. Clin. Oncol. 36 (15_Suppl. l), 11503. 10.1200/jco.2018.36.15_suppl.11503

[B7] Evans D. M. Sanerkin N. G. (1965). Primary leiomyosarcoma of bone. J. Pathol. Bacteriol. 90 (1), 348–350. 10.1002/path.1700900145 5843955

[B8] Feng X. Tonon L. Li H. Darbo E. Pleasance E. Macagno N. (2023). Comprehensive immune profiling unveils a subset of leiomyosarcoma with "hot" tumor immune microenvironment. Cancers (Basel) 15 (14), 3705. 10.3390/cancers15143705 37509366 PMC10378143

[B9] Fukumura D. Kloepper J. Amoozgar Z. Duda D. G. Jain R. K. (2018). Enhancing cancer immunotherapy using antiangiogenics: opportunities and challenges. Nat. Rev. Clin. Oncol. 15 (5), 325–340. 10.1038/nrclinonc.2018.29 29508855 PMC5921900

[B10] George S. Serrano C. Hensley M. L. Ray-Coquard I. (2018). Soft tissue and uterine leiomyosarcoma. J. Clin. Oncol. 36 (2), 144–150. 10.1200/JCO.2017.75.9845 29220301 PMC5759317

[B11] Guan Y. Feng D. Yin B. Li K. Wang J. (2022). Immune-related dissociated response as a specific atypical response pattern in solid tumors with immune checkpoint blockade. Ther. Adv. Med. Oncol. 14, 17588359221096877. 10.1177/17588359221096877 35547094 PMC9083034

[B12] Hua Y. Vella G. Rambow F. Allen E. Antoranz Martinez A. Duhamel M. (2022). Cancer immunotherapies transition endothelial cells into HEVs that generate TCF1(+) T lymphocyte niches through a feed-forward loop. Cancer Cell 40 (12), 1600–1618.e10. 10.1016/j.ccell.2022.11.002 36423635 PMC9899876

[B13] Humbert O. Cadour N. Paquet M. Schiappa R. Poudenx M. Chardin D. (2020). 18)FDG PET/CT in the early assessment of non-small cell lung cancer response to immunotherapy: frequency and clinical significance of atypical evolutive patterns. Eur. J. Nucl. Med. Mol. Imaging 47 (5), 1158–1167. 10.1007/s00259-019-04573-4 31760467

[B14] Isomoto K. Haratani K. Hayashi H. Shimizu S. Tomida S. Niwa T. (2020). Impact of EGFR-TKI treatment on the tumor immune microenvironment in EGFR mutation–positive non–small cell lung cancer. Clin. Cancer Res. 26 (8), 2037–2046. 10.1158/1078-0432.CCR-19-2027 31937613

[B15] Italiano A. Bessede A. Pulido M. Bompas E. Piperno-Neumann S. Chevreau C. (2022). Pembrolizumab in soft-tissue sarcomas with tertiary lymphoid structures: a phase 2 PEMBROSARC trial cohort. Nat. Med. 28 (6), 1199–1206. 10.1038/s41591-022-01821-3 35618839

[B16] Kim H. S. Cho H. J. Yun K.-H. Lee Y. H. Kim S. H. Baek W. (2021). Durvalumab and pazopanib in patients with advanced soft tissue sarcoma: a single-center, single-arm, phase 2 trial. J. Clin. Oncol. 39 (15_Suppl. l), 11551. 10.1200/jco.2021.39.15_suppl.11551

[B17] Kobayashi H. Zhang L. Hirai T. Tsuda Y. Ikegami M. Tanaka S. (2022). Comparison of clinical features and outcomes of patients with leiomyosarcoma of bone and soft tissue: a population-based cohort study. Jpn. J. Clin. Oncol. 52 (2), 143–150. 10.1093/jjco/hyab176 34791360

[B18] Lacuna K. Bose S. Ingham M. Schwartz G. (2023). Therapeutic advances in leiomyosarcoma. Front. Oncol. 13, 1149106. 10.3389/fonc.2023.1149106 36969049 PMC10031121

[B19] Lam T. C. Tsang K. C. Choi H. C. Lee V. H. Lam K. O. Chiang C. L. (2021). Combination atezolizumab, bevacizumab, pemetrexed and carboplatin for metastatic EGFR mutated NSCLC after TKI failure. Lung Cancer 159, 18–26. 10.1016/j.lungcan.2021.07.004 34303276

[B20] Lee W. S. Yang H. Chon H. J. Kim C. (2020). Combination of anti-angiogenic therapy and immune checkpoint blockade normalizes vascular-immune crosstalk to potentiate cancer immunity. Exp. Mol. Med. 52 (9), 1475–1485. 10.1038/s12276-020-00500-y 32913278 PMC8080646

[B21] Liniker E. Menzies A. M. Kong B. Y. Cooper A. Ramanujam S. Lo S. (2016). Activity and safety of radiotherapy with anti-PD-1 drug therapy in patients with metastatic melanoma. Oncoimmunology 5 (9), e1214788. 10.1080/2162402X.2016.1214788 27757312 PMC5048757

[B22] Liu J. Deng Y. T. Wu X. Jiang Y. (2021). Rechallenge with multi-targeted tyrosine kinase inhibitors in patients with advanced soft tissue sarcoma: a single-center experience. Cancer Manag. Res. 13, 2595–2601. 10.2147/CMAR.S300430 33776477 PMC7987272

[B23] Matushansky I. Charytonowicz E. Mills J. Siddiqi S. Hricik T. Cordon-Cardo C. (2009). MFH classification: differentiating undifferentiated pleomorphic sarcoma in the 21st Century. Expert Rev. Anticancer Ther. 9 (8), 1135–1144. 10.1586/era.09.76 19671033 PMC3000413

[B24] Meyer M. Seetharam M. (2019). First-line therapy for metastatic soft tissue sarcoma. Curr. Treat. Options Oncol. 20 (1), 6. 10.1007/s11864-019-0606-9 30675651

[B25] Miller K. D. Fidler-Benaoudia M. Keegan T. H. Hipp H. S. Jemal A. Siegel R. L. (2020). Cancer statistics for adolescents and young adults. CA Cancer J. Clin. 70 (6), 443–459. 10.3322/caac.21637 32940362

[B26] Novotny J. P. George S. (2021). Leiomyosarcoma: does location of primary help to determine the best systemic therapy options? Curr. Treat. Options Oncol. 22 (11), 99. 10.1007/s11864-021-00897-2 34524549

[B27] Pautier P. Italiano A. Piperno-Neumann S. Chevreau C. Penel N. Firmin N. (2022). Doxorubicin alone versus doxorubicin with trabectedin followed by trabectedin alone as first-line therapy for metastatic or unresectable leiomyosarcoma (LMS-04): a randomised, multicentre, open-label phase 3 trial. Lancet Oncol. 23 (8), 1044–1054. 10.1016/S1470-2045(22)00380-1 35835135

[B28] Rekhi B. Kaur A. Puri A. Desai S. Jambhekar N. A. (2011). Primary leiomyosarcoma of bone--a clinicopathologic study of 8 uncommon cases with immunohistochemical analysis and clinical outcomes. Ann. Diagn Pathol. 15 (3), 147–156. 10.1016/j.anndiagpath.2010.11.006 21393038

[B29] Roulleaux Dugage M. Nassif E. F. Italiano A. Bahleda R. (2021). Improving immunotherapy efficacy in soft-tissue sarcomas: a biomarker driven and histotype tailored review. Front. Immunol. 12, 775761. 10.3389/fimmu.2021.775761 34925348 PMC8678134

[B30] Sato Y. Morimoto T. Hara S. Nagata K. Hosoya K. Nakagawa A. (2021). Dissociated response and clinical benefit in patients treated with nivolumab monotherapy. Invest. New Drugs 39 (4), 1170–1178. 10.1007/s10637-021-01077-7 33566254

[B31] Schumacher T. N. Thommen D. S. (2022). Tertiary lymphoid structures in cancer. Science 375 (6576), eabf9419. 10.1126/science.abf9419 34990248

[B32] Sun H. Zhuang M. Cheng D. Zhu C. Liu Z. Qiu X. (2022). Primary leiomyosarcoma of cervical spine invading the vertebra without obvious osteoclasia: case report and literature review. J. Spinal Cord. Med. 45 (4), 643–647. 10.1080/10790268.2019.1656848 31539318 PMC9246264

[B33] Tawbi H. A. Burgess M. Bolejack V. Van Tine B. A. Schuetze S. M. Hu J. (2017). Pembrolizumab in advanced soft-tissue sarcoma and bone sarcoma (SARC028): a multicentre, two-cohort, single-arm, open-label, phase 2 trial. Lancet Oncol. 18 (11), 1493–1501. 10.1016/S1470-2045(17)30624-1 28988646 PMC7939029

[B34] Van Tine B. A. Eulo V. Toeniskoetter J. Ruff T. Luo J. Kemp L. (2023). Randomized phase II trial of cabozantinib combined with PD-1 and CTLA-4 inhibition versus cabozantinib in metastatic soft tissue sarcoma. J. Clin. Oncol. 41 (17_Suppl. l), LBA11504. LBA. 10.1200/jco.2023.41.17_suppl.lba11504

[B35] Wang B. Han Y. Zhang Y. Zhao Q. Wang H. Wei J. (2023). Overcoming acquired resistance to cancer immune checkpoint therapy: potential strategies based on molecular mechanisms. Cell Biosci. 13 (1), 120. 10.1186/s13578-023-01073-9 37386520 PMC10311815

[B36] Wang B. Liu J. Han Y. Deng Y. Li J. Jiang Y. (2022). The presence of tertiary lymphoid structures provides new insight into the clinicopathological features and prognosis of patients with breast cancer. Front. Immunol. 13, 868155. 10.3389/fimmu.2022.868155 35664009 PMC9161084

[B37] Wang G. Y. Lucas D. R. (2019). Primary leiomyosarcoma of bone: review and update. Arch. Pathol. Lab. Med. 143 (11), 1332–1337. 10.5858/arpa.2019-0375-RA 31661313

[B38] Wang Y. Yang L. Wang J. Gui L. Li W. Liu Z. (2021). Case report: first case of consolidation immunotherapy after definitive chemoradiotherapy in mediastinal lymph node metastatic sarcomatoid carcinoma. Front. Oncol. 11, 788856. 10.3389/fonc.2021.788856 35083145 PMC8785342

[B39] Wilky B. A. Trucco M. M. Subhawong T. K. Florou V. Park W. Kwon D. (2019). Axitinib plus pembrolizumab in patients with advanced sarcomas including alveolar soft-part sarcoma: a single-centre, single-arm, phase 2 trial. Lancet Oncol. 20 (6), 837–848. 10.1016/S1470-2045(19)30153-6 31078463

[B40] Wu Z. Cheng L. Cao Q. Ye S. Yu S. Sun M. (2022). Case report and literature review: primary leiomyosarcoma of the bone in the trochanteric region of the femur. Front. Surg. 9, 1045307. 10.3389/fsurg.2022.1045307 36704525 PMC9872517

[B41] You Y. Guo X. Zhuang R. Zhang C. Wang Z. Shen F. (2021). Activity of PD-1 inhibitor combined with anti-angiogenic therapy in advanced sarcoma: a single-center retrospective analysis. Front. Mol. Biosci. 8, 747650. 10.3389/fmolb.2021.747650 34869583 PMC8635153

[B42] Zhang J. Chen Y. Xing X. Wang Q. Liu K. Zhang E. (2022). Primary leiomyosarcoma of the spine: an analysis of imaging manifestations and clinicopathological findings. Insights Imaging 13 (1), 195. 10.1186/s13244-022-01336-y 36520263 PMC9755377

