# Supplementary figures and images for: Case report: the dissociated response and clinical benefit of primary leiomyosarcoma of the bone treated with penpulimab plus lenvatinib after failed multi-line therapy
Source: Front Pharmacol. 2023 Nov 9;14:1239699. doi: 10.3389/fphar.2023.1239699 (PMC10665504; doi:10.3389/fphar.2023.1239699)

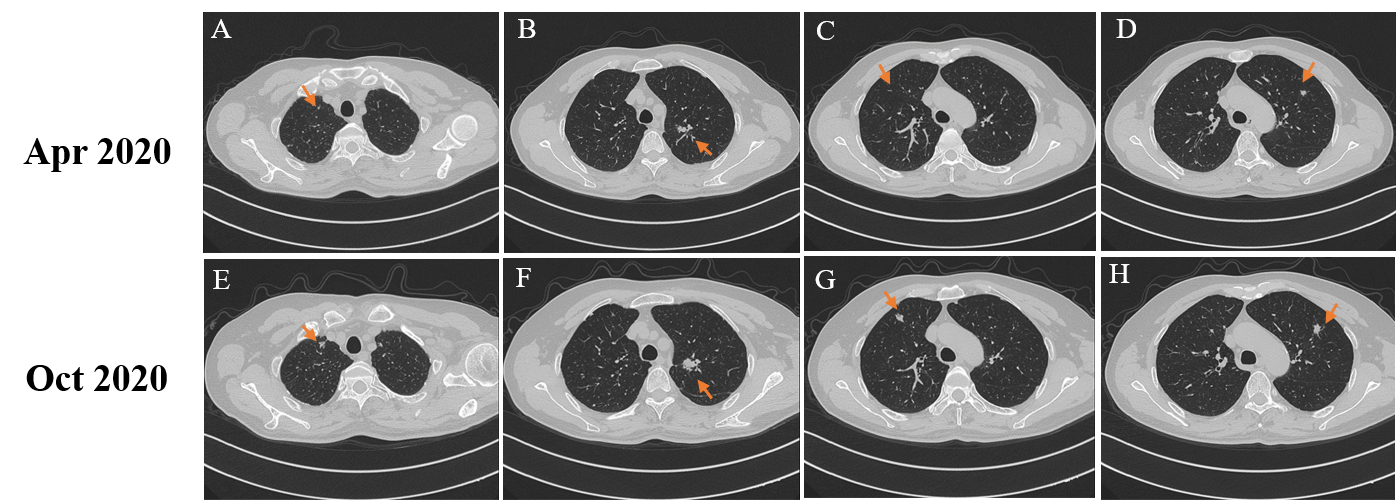

Supplement: Supplementary file 1 [file Image1.TIF]
